# Impact of Learning Methods on Spatial Knowledge Acquisition

**DOI:** 10.3389/fpsyg.2020.01322

**Published:** 2020-06-16

**Authors:** Xiaohe Qiu, Lala Wen, Changxu Wu, Zhen Yang, Qijun Wang, Hongting Li, Duming Wang

**Affiliations:** ^1^Department of Psychology, Zhejiang Sci-Tech University, Hangzhou, China; ^2^Department of Systems and Industrial Engineering, College of Engineering, University of Arizona, Tucson, AZ, United States

**Keywords:** spatial knowledge, learning methods, field navigation learning, 2D map-assisted field navigation learning, virtual street view map learning, active learning, passive learning

## Abstract

Research on the acquisition of spatial knowledge not only enriches our understanding of the theory of spatial knowledge representation but also creates practical value for the application of spatial knowledge. The aim of this study is to understand the impact of different learning methods on the acquisition of spatial knowledge, including the role of 2D maps, the difference between physical interaction and virtual interaction, and whether passive learning can replace active learning in virtual environments. One experiment was conducted, in which landmark knowledge and configurational knowledge were measured. Results indicate that 2D maps play a supporting role in acquiring both landmark knowledge and configurational knowledge. In addition, physical learning was associated with better spatial knowledge representation compared with virtual learning. An analysis of observational data in the third comparison found no significant difference between passive learning and active learning using virtual street view maps. However, with high-quality learning materials, passive learning can contribute to the acquisition of spatial knowledge more efficiently than active learning.

## Introduction

The importance of wayfinding is obvious in everyday life. Wayfinding involves multiple cognitive processes, such as encoding environmental characteristics, building and maintaining spatial representations in memory, and using them (Piccardi et al., 2019). During wayfinding, one acquires knowledge of the environmental space and stores it in his way, forms a “cognitive map” of the environment, and finds ways based on it (Downs and Stea, 1974; Golledge, 1999). Construction of cognitive map involves the acquisition of spatial knowledge.

Spatial knowledge includes the knowledge of the spatial relationships between objects of an environment in the individual’s wayfinding process (declarative spatial knowledge) and the knowledge about how to move in this environment (procedural spatial knowledge) (Sorrentino et al., 2019). The key to solving spatial problems lies in whether the individual can efficiently acquire spatial knowledge, that is, forming a complete spatial environmental representation as well as saving time and cognitive sources.

Spatial learning cannot be separated from learning methods. In this study, we focus on how individuals build spatial knowledge and what impacts different learning methods have on the acquisition of spatial knowledge.

### Spatial Knowledge

As a representation of one’s environment, spatial knowledge contains environmental characteristics, the relative position between objects, and properties of spatial relationships (Lynch, 1960; Kuipers, 1982; Papadopoulos et al., 2017a). With respect to the development of spatial knowledge, Siegel and White (1975) constructed the landmark–route–survey (LRS) model, which includes three steps in the process of knowledge acquisition: landmark knowledge, route knowledge, and survey knowledge. On the basis of this model, many researchers (Thorndyke and Hayes-Roth, 1982; Gale et al., 1990; Tversky et al., 1994; Darken and Sibert, 1996; Elvins, 1997; Ishikawa and Montello, 2006; Spiers and Maguire, 2008) posit that cognitive maps, as a representation of spatial knowledge, must comprise landmark knowledge, route knowledge, and survey knowledge.

Per the LRS model, spatial knowledge is specifically believed to consist of landmark knowledge and configurational knowledge (Satalich, 1995). To measure the former, researchers employed scene recognition and wayfinding performance metrics; to quantify the latter, they utilized measures of distance and direction between locations, relative positions, and other aspects of the overall environmental representation. De Goede and Postma (2015) designed scene recognition tasks for landmark knowledge; the participants needed to determine whether the scene presented to them was from the city in which they lived and then complete a five-point familiarity rating task. For configurational knowledge, De Goede and Postma (2015) facilitated distance comparison and relative position tasks. In the distance comparison tasks, two pairs of locations appeared, and the participants had to indicate which pair was most representative of real-world locations. In the relative position tasks, participants were shown triads of places that were either in their correct relative spatial positions or that were incorrect mirror images of their correct spatial positions. They had to judge whether each triad accurately depicted the relative physical position of actual places in the physical environment. In short, De Goede and Postma’s paradigm can measure landmark knowledge and configurational knowledge simultaneously, which is meaningful to the comprehensive measurement of spatial knowledge. Through exhaustive assessment, researchers may achieve greater insight into the differences between the components of spatial representation.

### Learning Methods

Different learning methods have different effects on the acquisition and representation of spatial knowledge. The most commonly used methods are navigation learning, map learning, virtual field navigation learning, and description learning. Map learning can be further classed into traditional paper map learning, electronic 2D map learning, and 3D street view map learning according to the different learning materials. Some interactive street view maps are also considered a form of virtual learning.

The acquisition of spatial knowledge via field navigation learning occurs by walking in a new environment, as people gain procedural knowledge of routes connecting diverse locations. Its greatest advantage is the gaining of environmental details through physical interaction, particularly non-visual channels (hearing, touch, smell, proprioception, etc.) (Riecke et al., 2010; Ruddle et al., 2011). According to Siegel’s spatial knowledge model, with the richer experience of living in the environment, spatial representation gradually develops from inaccurate landmark knowledge to route knowledge and eventually forms survey knowledge (Siegel and White, 1975). However, some researchers also believe that a configurational representation of the environment cannot be formed through field navigation learning alone. Thorndyke and Hayes-Roth (1982) compared the impact of field navigation learning and map learning on task completion. They found that with extensive exposure (months of learning), learners still had great difficulty completing non-self-centered perspective tasks (e.g., locating the relative location of a room on the basis of spatial references of two places). Moeser (1988) studied student nurses’ representations of a hospital’s layout and found no difference in spatial representation between nurses who worked in the hospital for one year and nurses who worked there for three years. Both groups of nurses ultimately provided landmark or route representations that were somewhat inaccurate. Ishikawa and Montello (2006) found that some participants, regardless of how many times they repeated the route they were learning, performed badly on tasks such as direction and distance estimation, and their performance did not improve with practice. These results illustrate that field navigation learning does not necessarily promote an accurate representation of survey knowledge.

Maps provide comprehensive information about a reader’s environment, thereby facilitating simultaneous working memory processing (Coluccia et al., 2007; Blalock and Clegg, 2010) and acquisition of survey knowledge. This feature enables learners to gain a more complete representation of the environment’s global layout (Meilinger et al., 2013). However, this type of learning is easily affected by a map’s medium; studies show that traditional paper maps are more conducive to the acquisition of spatial knowledge than electronic 2D maps (Ishikawa et al., 2008; Dickmann, 2012).

Virtual environments (VEs) depict visuospatial information from a 3D first-person perspective (Richardson et al., 1999; Jansen-Osmann, 2002). Successful learning in VEs can be applied to physical environments (Ruddle et al., 1997; Jacobs et al., 1998) and predict wayfinding performance within them (Coutrot et al., 2019). Abundant research demonstrates that virtual field navigation learning is not only beneficial to wayfinding performance but also enhances spatial representation and further helps create a better-quality cognitive map (Ruddle et al., 1997; Ohuchi et al., 2006; Lahav and Mioduser, 2008).

However, some studies have shown that virtual field navigation learning is not effective in assisting learners to develop spatial layout representation (Münzer et al., 2006; Ishikawa et al., 2008). Spatial representation obtained by single virtual field navigation learning has some limitations. Ruddle et al. (1997) found that increased practice time did not trigger an improvement in task performance with a non-self-centered perspective. Münzer et al. (2006) also found that additional training in VEs had no significant effect on the acquisition of spatial knowledge.

3D street view maps provide 360-degree spatial representation, including more information about the surroundings of a particular location than do 2D maps (Gu, 2018). Street view map learning can also be regarded as a manner of engaging in virtual field navigation learning; it takes less time than field navigation learning. Compared to street view maps, VEs place the learner in an immersive environment that is more similar to the physical environment, which provides more realistic spatial information and is thus more intuitive than street view maps (Gu, 2018). Unlike 2D maps, street view maps and VEs change learners’ perspective from a bird’s-eye view to a pedestrian view, mimicking an embodied perspective, and provide a more realistic image with which to interact (König et al., 2019). Therefore, with appropriate cautions, research on street view map learning can be conducted with reference to VE studies. Moreover, 2D maps can be added to street view maps to assist learners in forming configurational representations. The biggest disadvantage to street view map learning is the delay in learning that most users experience, which is a challenge to cognitive load.

In VEs, human–computer interaction is virtual. This kind of learning can be either passive or active. Active learning usually means that participants can control their will and movement to learn, while passive learning provides limited exposure to the material via videos, slides, and other visual modes with limited or no cognitive control on the part of the learner (Chrastil and Warren, 2013). Active learning consists of cognitive and physical components; cognitive components include mental manipulation of spatial information, attention distribution, and route decision-making, while physical components comprise motion control, proprioception information, and vestibular information (Chrastil and Warren, 2012). These components work synergistically in spatial task performance (Picucci et al., 2013; Larrue et al., 2014; Gardony et al., 2015; Rand et al., 2015). Appleyard (1970) was one of the first researchers to propose that bus drivers and passengers have distinct differences in acquiring spatial knowledge, thereby leading to a series of studies on the impact of active learning and passive learning on spatial knowledge acquisition. The difference between active learning and passive learning not only affects the cognitive structure of the environment but also influences learners’ visual memory of the environment (Afrooz et al., 2018). Active exploration of a new environment will likely lead to better spatial learning than passive exposure will (Chrastil and Warren, 2015). The memory advantage of active learning is called the subject-performed task (SPT) effect (Brooks, 1999; Sauzéon et al., 2012). It describes superior memory performance patterns, especially when coding involves the learner directly performing learning actions rather than observing the actions of others that may be related to remembered words (Zimmer and Engelkamp, 1999). Chrastil and Warren (2013) found that participants who had the opportunity to decide the route they would follow in spatial learning outperformed those who watched a wayfinding video depicting that route. Meade et al. (2019) found that active learning can improve the accuracy of spatial memory in VEs more so than passive learning. Many researchers have found that the mental impact of active learning on spatial information that the working memory retains contributes to the acquisition of route knowledge and survey knowledge (Münzer et al., 2006; Labate et al., 2014; Weisberg and Newcombe, 2016; Blacker et al., 2017).

Still, some studies show that active learning and passive learning have less impact on the acquisition of spatial knowledge than other kinds of learning. Allison and Redhead (2017) found that participants with navigation experience, whether they actively explored their environment or passively watched videos, could maintain their orientation and spatial updating in VEs, but that non-experienced participants were unable to maintain their orientation, even if they actively explored VEs. Cao et al. (2019) found that compared with those who explored a virtual museum passively, participants who explored the same museum actively traveled longer in completing the egress task. Christou and Bülthoff (1999) conducted a similar study but found no difference in the scene recognition performance of active learning groups and passive learning groups. While active exploration of a new environment may seem more effective than passive learning (Chrastil and Warren, 2015), current literature does not provide a definitive answer. The difference in effectiveness between active learning and passive learning of street view maps still needs to be determined by empirical research.

### Research Goal

At present, research relative to map learning methods is mostly concentrated around the use of traditional paper maps. Although many studies in the area of virtual field navigation learning are emerging, research on some learning methods, such as 3D street maps, is still lacking. Unfortunately, the literature that is emerging around learning methods is inconsistent. In terms of map learning and field navigation learning, many studies assert that map learning is an important way to form survey knowledge, while field navigation learning can help form route knowledge. Conclusions as to whether long-term field navigation learning can assist in forming survey knowledge are inconsistent. The ability of virtual field navigation learning to replace field navigation learning is currently in dispute. One possible reason lies in the inconsistency between the way in which the composition and paradigm of spatial knowledge are measured in many studies. Previous research that investigated subjects’ performance in wayfinding tasks mainly focused on route knowledge, that is to say, different spatial knowledge components have seldom been measured comprehensively. At this point, the impact of active learning and passive learning, especially for 3D street view maps, on the acquisition of spatial knowledge is arguably of a high priority for researchers to examine.

Previous studies (Haq and Girotto, 2003; Montello et al., 2004; Isaacson and Shoval, 2006) were concerned with (1) visual learning methods (field-based, map-based, and virtual-based), (2) non-visual learning methods (e.g., audio, tactile, and haptic), and (3) an arbitrary combination of these two kinds. Non-visual learning methods such as audio learning are mainly used as the most common alternative approaches to meet the particular needs of learners (e.g., vision-impaired persons) (Szymczak et al., 2012; Yu and Habel, 2012; Papadopoulos et al., 2017b). Our main research purpose is to explore the impact of different learning methods on spatial learning in a large-scale environment. Thus, we consider that the non-visual learning methods are not suitable. Although audio non-visual learning methods can reduce the load on visual attention when combined with visual learning methods, no conclusions for a single learning kind can be drawn when combining more than one method from other kinds. Moreover, controlling when the non-visual information presents and how long it lasts is difficult because it is very different from visual information. Therefore, comparing spatial knowledge acquisition among the common learning methods such as field navigation, map learning, and virtual field navigation learning is still considered an open issue.

Thus, this study aims to explore the influences of four learning methods (i.e., navigation learning, 2D map-assisted navigation learning, 2D map-assisted street view map active learning, and 2D map-assisted street view map passive learning) on landmark knowledge and configurational knowledge. Moreover, the main differences among these four learning methods were attributed to the following three independent variables: the assistance of 2D maps, interaction mode, and learning mode. Therefore, three interested pairwise comparisons were further conducted.

(1) The role of 2D maps in the process of acquiring spatial knowledge was investigated by comparing spatial knowledge learning with field navigation and 2D map-assisted field navigation.
(2) Differences between virtual interaction and physical interaction were investigated to understand whether and to what extent can physical interaction be replaced by virtual interaction. Thus, differences in spatial knowledge learning between 2D map-assisted field navigation (i.e., physical interaction) and active learning using street view map (i.e., virtual interaction) were compared.
(3) The influence of interaction modes (active learning or passive learning) in street view map learning on the acquisition of spatial knowledge was investigated.

## Materials and Methods

### Participants

Participants included a total of 120 college students (60 males and 60 females) who had no experience relative to the experimental area in the month immediately prior to the study and were generally unfamiliar with the area. Participants ranged in age from 17 to 24 years old (*M* = 20.8; *SD* = 1.5). All participants were assigned randomly to one of the four learning method conditions.

This study was approved by Institutional Review Board of the Zhejiang Sci-Tech University. All subjects gave written informed consent in accordance with the Declaration of Helsinki.

### Design

This study employed a between-subjects design. The learning method served as the independent variable. The four learning method conditions were the field navigation learning group, the 2D map-assisted field navigation learning group, the 2D map-assisted street view map active learning group, and the 2D map-assisted street view map passive learning group. Dependent variables included accuracy, reaction time (RT) and confidence ratings of the scene recognition task, orientation judgment task, and configurational representation task. Distance perception representation task was an estimation task without absolute right or wrong answers. Thus, we used the absolute difference of distance (compared with the correct distance) to substitute for the accuracy in this task. RT and confidence ratings were also measured in the distance perception representation task.

### Environment and Materials

#### Environment

**FIGURE 1:**
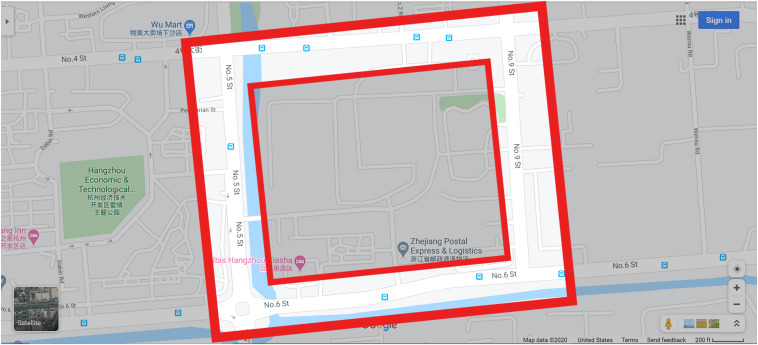
The experimental area.

The experimental area (highlighted area, Figure 1) included No. 4 Ave., No. 9 Ave., No. 6 Ave., and No. 5 Ave., Xiasha, Hangzhou, which does not include the inner area.

**FIGURE 2:**
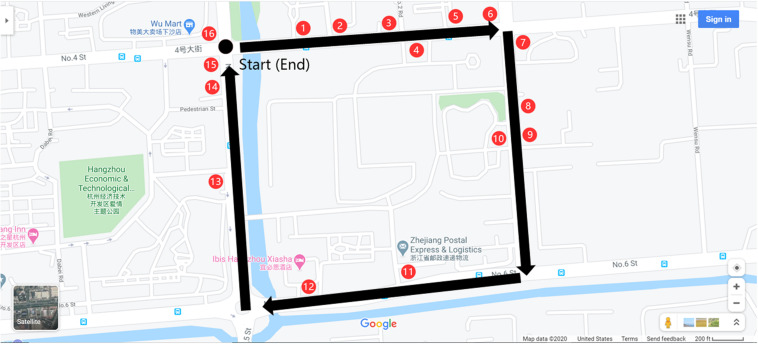
The path through the environment is identified by the superimposed black arrows, and the 16 red ovals shows each landmark’s position (they are not present during viewing).

**FIGURE 3:**
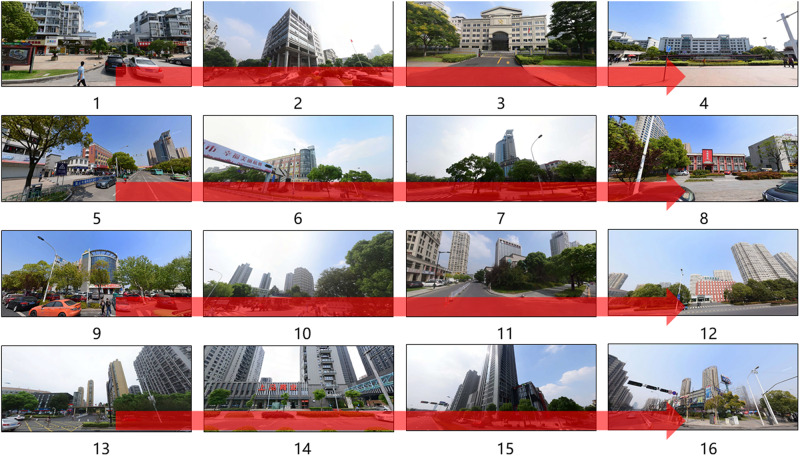
The participants start from the starting point (see Figure 2), walk by 16 landmarks and back to the starting point (the ending point).

The experimental area is approximately 500 m × 500 m with a mass of shops, businesses, bars, and restaurants. It contained 16 large landmarks (used as task materials), 12 buildings in diverse styles, and fixed features such as road crossings, car traffic, and pedestrian zones (see Figures 2, 3). This experimental area can be well explored within half an hour. Compared with many physical environments used for spatial navigation research (Krüger et al., 2004; Ishikawa et al., 2008; Willis et al., 2009), the experimental area selected in the present study is relatively larger and more complex. Participants needed to begin at the starting point and proceed clockwise in a square road around the area to the ending point (identical to the starting point), with four turns within 25 min.

#### Materials

**FIGURE 4:**
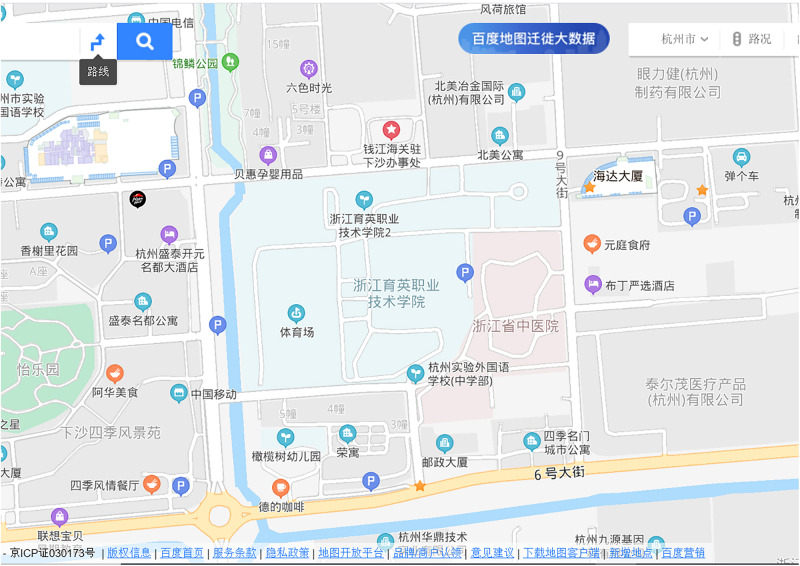
The assisted 2D map.

**FIGURE 5:**
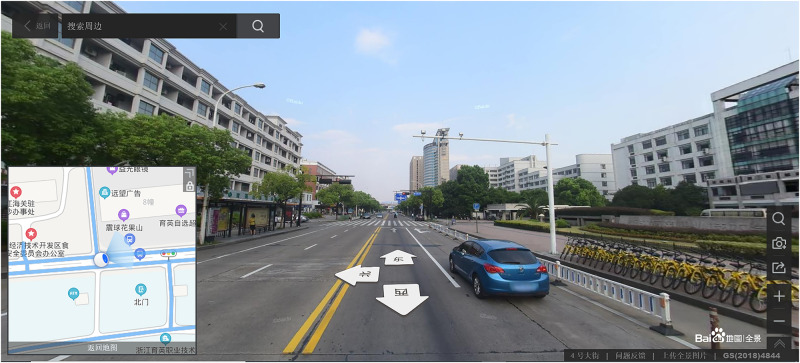
One screenshot of displays of street view map learning.

For the assisting map, which was shown to the 2D map-assisted field navigation learning group, the printed map (Figure 4) was A4-sized (1:50) and had a resolution of 2570 × 1817 pixels. The street view map (Figure 5)^1^ was drawn from Baidu Map, a desktop and mobile web mapping service application. A video exploring the environment actively from a first-person perspective was provided for 2D map-assisted street view map learning (passive). The production method of the video is as follows: One researcher completed a simulated navigation task in strict accordance with instructions for the task. At the same time, every action was captured by a screen recorder software and then edited into a 25 min video.

**FIGURE 6:**
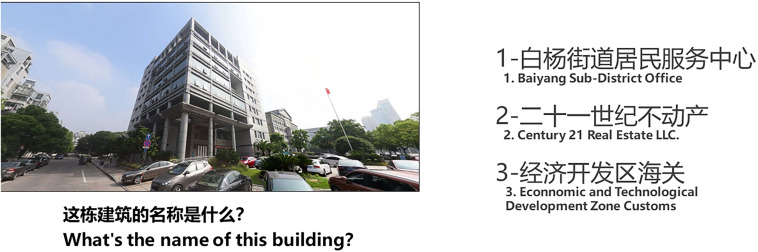
Scene recognition task: Task display.

**FIGURE 7:**
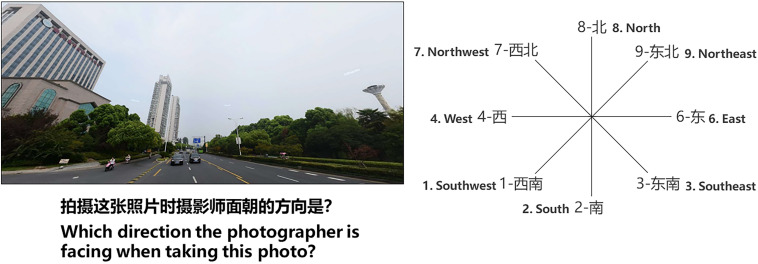
Orientation judgment task: Task display.

**FIGURE 8:**
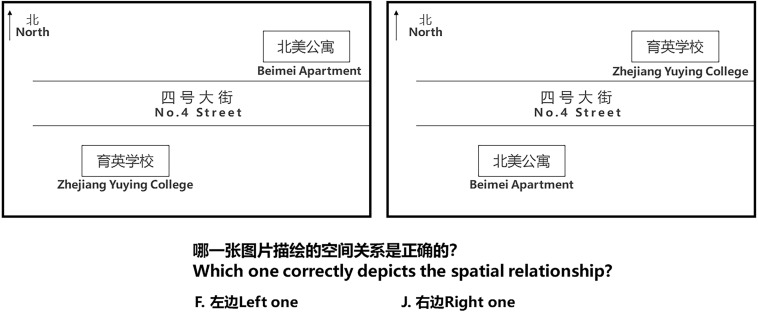
Configurational representation task: Task display.

**FIGURE 9:**
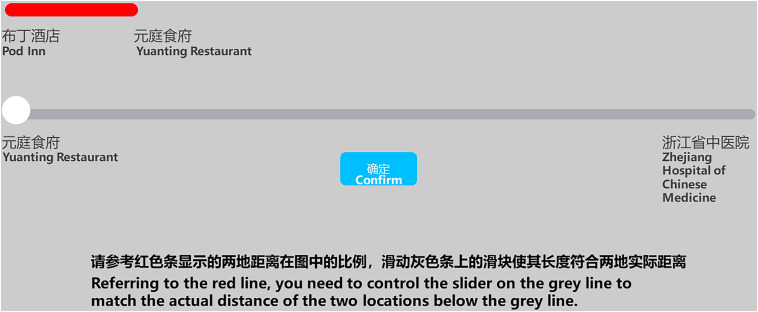
Distance perception representation task: Task display.

Spatial knowledge is composed of landmark knowledge and configurational knowledge. Landmark knowledge is measured by scene recognition tasks (Figure 6), and configurational knowledge is measured by orientation judgment tasks (Figure 7), configurational representation tasks (Figure 8), and distance perception representation tasks (Figure 9).

**FIGURE 10:**
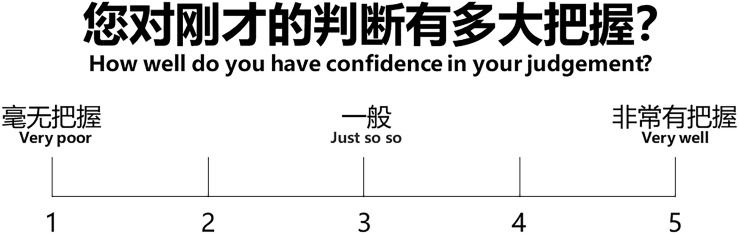
Rating display.

The scene recognition task (Figure 6) primarily measured the quality of the participants’ landmark knowledge. The task consisted of 17 trials. The participants were shown a picture of a building in the learning area on the left, with options for building names on the right. They had to recall the name of the building and press the corresponding number key to identify its name as quickly as they could. The participants were then asked how confident they were in their judgment, indicated on a scale from 1 (not confident) to 5 (very confident) (Figure 10).

Scene recognition and orientation judgment tasks (Figure 7) were conducted with the aim to test configurational knowledge. Sixteen trials were carried out in this task. First, a picture of a building in the learning area was presented on the left, with a direction comparison picture presented on the right. The participants were instructed to identify the scene, identify from what direction they were facing the scene, and press the corresponding number key to identify that direction as quickly as they could. The confidence rating display (consistent with the rating display given in the scene recognition task, Figure 10) was provided and remained on the screen until the subject responded. This task and the scene recognition task both measured scene recognition knowledge; thus, they were presented to participants in random order.

The configurational representation task (Figure 8) was preceded by an instructional session and 20 trials, containing two pictures of the layout of buildings in the learning area (one being correct, the other being incorrect). The participants were asked to judge which picture accurately depicted the relative physical position of the places in the physical environment. The confidence rating display was presented again, as it was in the scene recognition task.

In the last test, the distance perception task (Figure 9), the participants completed 20 trials. First, a picture was shown on the screen, containing two segments. The upper segment (red) represented the distance between two reference points on a graph, and the lower line contained a slider that could be dragged left or right by the participant. In correspondence to the upper line, the participant controlled the slider by using the mouse to match the actual distance of the two test locations in the graph. After they pressed the “confirm” button, the same confidence rating display was shown.

The study used a laptop (Asus A43S, 1366 × 768 pixels), with a keyboard (Logitech K260) and a mouse. The experimental program was developed by E-prime 2.0 and JavaScript to present stimuli and collect data.

### Procedure

The participants were given the instructions relevant to their experimental condition and asked to read them carefully. The 2D map-assisted street view map active learning group also needed to familiarize themselves with the street view navigation system before the start of the experiment tasks. Once the experimenter verified that participants understood the tasks and interacted properly with the navigation system, the learning tasks began.

The field navigation learning group followed the experimenter to learn the experimental environment. The 2D map-assisted field navigation learning group was provided with a paper map (Figure 4) relative to the learning area within the environment and followed the experimenter. Walking in the street view environment, the street view map active learning group freely explored it until they reached the next intersection, when the experimenter told the participants to which direction they should turn to avoid different wondering or learning time. The 2D street view map passive learning group watched the video in a quiet, non-experimental area.

During the learning process, the experimenter did not communicate with the participants and trained only one participant each time. The participants completed the learning session within 25 ± 1.5 min, and then rested 10 ± 2 min before returning to the lab for the test. Finally, after completing the scene recognition and orientation judgment tasks, each participant performed the configurational representation and distance perception representation tasks.

## Results

Although different dependent variables might be correlated with each other, the pre-data analysis showed that MANOVA was not suitable for this study because of the following reasons: (1) The dependent variables were not uniform for the four spatial knowledge tasks. For the distance perception representation task, the absolute difference of distance was used to substitute for the accuracy. (2) The RT of the distance perception representation task was markedly larger than those of the other three tasks, which resulted in an extremely skewed distribution of the RT. This condition may have violated the premise of a normal distribution and led to a positive result of the Box’s M test when we used MANOVA to analyze the two uniform dependent variables (i.e., RT and confidence rating) among four spatial knowledge tasks [*F*_(__45_,_322_,_162__)_ = 23.18, *p* < 0.001].

Thus, we first performed single-factor ANOVA to explore the impact of the learning methods on performance in each spatial knowledge task. After that, three interested pairwise comparisons were conducted to investigate the potential influence of the 2D map, the physical/virtual interaction mode, and the active/passive learning mode on the performance in spatial knowledge measures.

IBM SPSS Statistics 21 was used to analyze the experimental data.

### Integrated Analysis of Each Dependent Measure

The results of descriptive statistics and ANOVAs are shown in Table 1. All the results of *post hoc* tests were Bonferroni corrected.

**TABLE 1:** Differences among learning methods in terms of performance on different spatial knowledge tasks (Mean ± SE).

	Field navigation learning (*n* = 30)	2D map-assisted field navigation learning (*n* = 30)	Street view map active learning (*n* = 30)	Street view map passive learning (*n* = 30)	*F*
**Scene recognition task**					
Accuracy	0.67 ± 0.02	0.81 ± 0.02	0.71 ± 0.02	0.78 ± 0.02	7.877***
Reaction time (s)	9.47 ± 0.43	8.96 ± 0.47	10.43 ± 0.63	8.74 ± 0.43	2.280
Confidence ratings	3.87 ± 0.07	4.41 ± 0.08	3.96 ± 0.11	4.19 ± 0.12	6.227**
**Orientation judgment task**					
Accuracy	0.44 ± 0.04	0.65 ± 0.03	0.41 ± 0.03	0.46 ± 0.04	10.401***
Reaction time (s)	14.50 ± 1.00	15.08 ± 0.84	15.43 ± 1.01	17.51 ± 1.13	1.734
Confidence ratings	3.39 ± 0.16	3.79 ± 0.12	3.33 ± 0.17	3.25 ± 0.16	2.454
**Configurational representation task**					
Accuracy	0.80 ± 0.03	0.88 ± 0.02	0.74 ± 0.03	0.78 ± 0.03	4.575**
Reaction time (s)	8.95 ± 0.70	8.00 ± 0.51	9.71 ± 0.89	9.15 ± 0.69	1.024
Confidence ratings	4.27 ± 0.14	4.60 ± 0.09	3.92 ± 0.15	4.37 ± 0.12	4.744**
**Distance perception representation task**					
Absolute difference of distance (cm)	8.31 ± 0.29	7.79 ± 0.25	8.49 ± 0.19	8.34 ± 0.28	1.406
Reaction time (s)	151.48 ± 6.50	159.17 ± 7.85	150.09 ± 7.52	150.38 ± 6.82	0.358
Confidence ratings	2.85 ± 0.10	3.24 ± 0.10	2.80 ± 0.16	3.00 ± 0.15	2.247

**p < 0.01, ***p < 0.001. F indicates the F-value of the main effect of learning methods.

#### Scene Recognition Task

**FIGURE 11:**
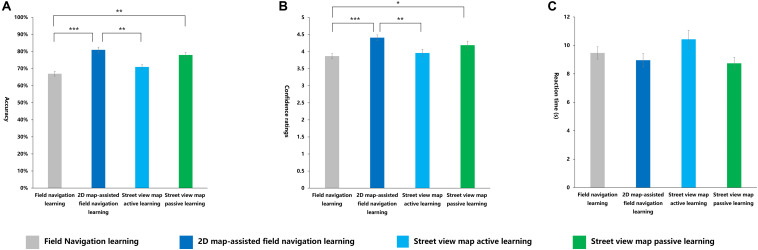
Indexes in scene recognition task under four learning methods. **(A)** Accuracy, **(B)** confidence ratings, **(C)** reaction time (s). *p < 0.05; **p < 0.01; ***p < 0.001.

A significant main effect of learning methods was observed for accuracy [*F*_(__3_,_116__)_ = 7.877, *p* < 0.001, η^2^*_p_* = 0.169]. The *post hoc* tests revealed that the average accuracy of the 2D map-assisted field navigation learning group was significantly higher than that of the field navigation learning group (*p* < 0.001). The average accuracy of the street view map passive learning group was significantly higher than that of the field navigation learning group (*p* < 0.01). The average accuracy of the 2D map-assisted field navigation learning group was significantly higher than that of the street view map active learning group (*p* < 0.01). No other significant effects were observed (Figure 11A). The following equation roughly shows the relationship: 2D map-assisted field navigation learning ≃ street view map passive learning > street view map active learning ≃ field navigation learning.

Learning methods had a significant main effect on confidence ratings [*F*_(__3_,_116__)_ = 6.227, *p* < 0.01, η^2^*_p_* = 0.139]. The *post hoc* tests demonstrated that the confidence ratings of the 2D map-assisted field navigation learning group were significantly higher than those of the field navigation learning group (*p* < 0.001). The street view map passive learning group had significantly higher scores in judgment confidence compared with the field navigation learning group (*p* < 0.05). Compared with the street view map active learning group, the 2D map-assisted field navigation learning group showed higher confidence scores (*p* < 0.01). No other significant effects were observed (Figure 11B). The following equation roughly shows a similar relationship: 2D map-assisted field navigation learning ≃ street view map passive learning > street view map active learning ≃ field navigation learning.

No significant differences in RT were observed among different learning methods (Figure 11C).

#### Orientation Judgment Task

**FIGURE 12:**
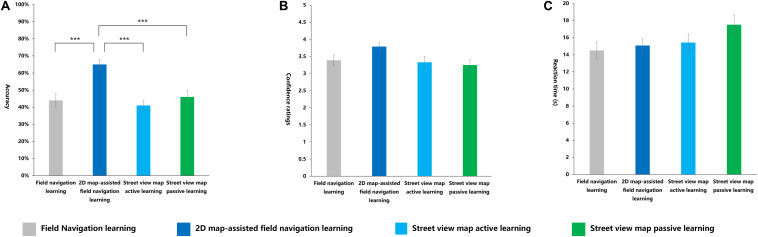
Indexes in orientation judgment task under four learning methods. **(A)** Accuracy, **(B)** confidence ratings, **(C)** reaction time (s). ***p < 0.001.

The main effect of the learning methods was significant [*F*_(__3_,_116__)_ = 10.401, *p* < 0.001, η^2^*_p_* = 0.212]. The *post hoc* tests showed that the average accuracy of the 2D map-assisted field navigation learning group was significantly higher than that of the field navigation learning group (*p* < 0.001), the street view map active learning group (*p* < 0.001), and the street view map passive learning group (*p* < 0.001). No significant differences were observed among the field navigation learning group, the street view map active learning group, and the street view map passive learning group (Figure 12A). The following equation roughly shows the relationship: 2D map-assisted field navigation learning > street view map passive learning ≃ field navigation learning ≃ street view map active learning.

No significant differences were observed in the confidence ratings and RT among all groups (Figures 12B,C).

#### Configurational Representation Task

**FIGURE 13:**
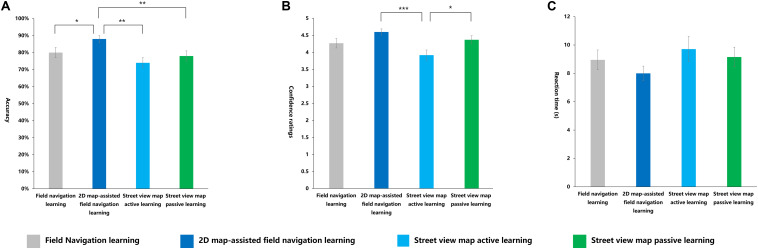
Indexes in configurational representation task under four learning methods. **(A)** Accuracy, **(B)** confidence ratings, **(C)** reaction time (s). *p < 0.05; **p < 0.01; ***p < 0.001.

The main effect of the learning methods was significant [*F*(3, 116) = 4.575, *p* < 0.01, η^2^*_p_* = 0.106]. The *post hoc* test results showed that the average accuracy of the 2D map-assisted field navigation learning group was significantly higher than that of the field navigation learning group (*p* < 0.05), the street view map active learning group (*p* < 0.01), and the street view map passive learning group (*p* < 0.01). No significant difference in accuracy was observed among the field navigation learning group, the street view map active learning group, and the street view map passive learning group (Figure 13A). The following equation roughly shows the relationship: 2D map-assisted field navigation learning > field navigation learning ≃ street view map passive learning ≃ street view map active learning.

In terms of confidence ratings, ANOVA revealed that the main effect of the learning methods was significant [*F*_(__3_,_116__)_ = 4.744, *p* < 0.01, η^2^*_p_* = 0.109]. The *post hoc* test showed that the confidence ratings of the 2D map-assisted field navigation learning group and the street view map passive learning group were significantly higher than that of the street view map active learning group (*p* < 0.001; *p* < 0.05). No other significant effects were observed (Figure 13B). The following equation roughly shows the relationship: 2D map-assisted field navigation learning ≃ street view map passive learning > field navigation learning ≃ street view map active learning.

No significant differences in RT were observed among the different learning methods (Figure 13C).

#### Distance Perception Representation Task

**FIGURE 14:**
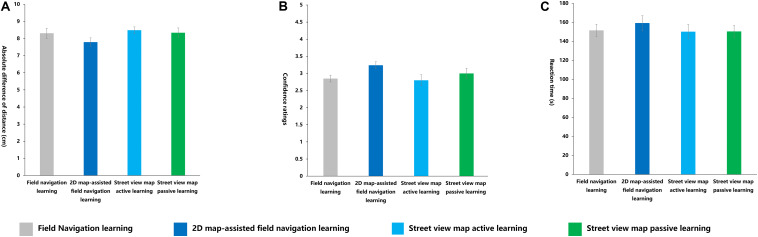
Indexes in distance perception representation task under four learning methods. **(A)** Accuracy, **(B)** confidence ratings, **(C)** reaction time (s).

No main effect of different learning methods was observed on the absolute difference of distance, confidence ratings, and RT in the distance perception representation task (Figure 14).

### Interested Pairwise Comparisons

#### 2D Map-Assisted Learning Versus Unassisted Learning

The main purpose of the comparison of the performance of the field navigation learning group and the 2D map-assisted field navigation learning group is to investigate the role of 2D maps in the process of acquiring spatial knowledge.

In terms of accuracy, the 2D map-assisted field navigation learning group showed significantly higher scores than the field navigation learning group [*t*_(__58__)_ = 4.829, *p* < 0.001, *d* = 1.25; *t*_(__58__)_ = 4.829, *p* < 0.001, *d* = 1.25; *t*_(__58__)_ = 2.413, *p* < 0.05, *d* = 0.62]. No significant differences were observed in terms of RT between the two groups [*t*_(__58__)_ = 0.447, *p* > 0.05, *d* = 0.11; *t*_(__58__)_ = 0.447, *p* > 0.05, *d* = 0.11; *t*_(__58__)_ = 1.114, *p* > 0.05, *d* = 0.29; *t*_(__58__)_ = −0.755, *p* > 0.05, *d* = 0.19]. Regarding the subjective evaluation of the participants in the tasks, the 2D map-assisted field navigation learning group showed significantly higher scores in judgment confidence compared with the field navigation learning group [*t*_(__58__)_ = −2.002, *p* = 0.050, *d* = 0.52; *t*_(__58__)_ = −2.002, *p* = 0.050, *d* = 0.52; *t*_(__58__)_ = −1.941, *p* = 0.057 > 0.050, *d* = 0.50; *t*_(__58__)_ = −2.763, *p* < 0.01, *d* = 0.7]. However, absolute difference of distance showed no significant differences in the distance perception representation task [*t*_(__58__)_ = 1.356, *p* > 0.05, *d* = 0.35].

#### Virtual Learning Versus Field Navigation Learning

Differences in spatial knowledge acquisition between the 2D map-assisted field navigation group (i.e., physical interaction) and the 2D map-assisted street view map learning group (active) (i.e., virtual interaction) were compared to examine whether and to what extent physical interaction can be replaced by virtual interaction.

Significant differences were observed in terms of accuracy between the 2D map-assisted field navigation learning group and the street view map learning, and the former performed better than the latter [*t*_(__58__)_ = 3.240, *p* < 0.001, *d* = 0.84; *t*_(__58__)_ = 5.555, *p* < 0.001, *d* = 1.43; *t*_(__58__)_ = 4.018, *p* < 0.05, *d* = 0.62]. In terms of RT, no significant differences were found between the two groups [*t*_(__58__)_ = −1.755, *p* > 0.05, *d* = 0.46; *t*_(__58__)_ = 0.118, *p* > 0.03, *d* = 0.11; *t*_(__58__)_ = −1.680, *p* > 0.05, *d* = 0.43; *t*_(__58__)_ = 0.835, *p* > 0.05, *d* = 0.22]. With respect to confidence ratings, the 2D map-assisted field navigation learning group had significantly higher scores in judgment confidence compared with the street view map learning group [*t*(58) = 3.346, *p* < 0.01, *d* = 0.86; *t*(58) = 2.203, *p* < 0.05, *d* = 0.57; *t*(58) = 3.763, *p* < 0.001, *d* = 0.97; *t*(58) = 2.299, *p* < 0.05, *d* = 0.59]. A significant effect was also found in the absolute difference for the distance task [*t*_(__58__)_ = −2.227, *p* < 0.05, *d* = 0.57], with a lower error in the 2D map-assisted field navigation learning group.

#### Passive Learning Versus Active Learning

The influence of learning modes (active learning or passive learning) in street view map learning on spatial knowledge acquisition was investigated by comparing the 2D map-assisted street view map learning group (active) and the 2D map-assisted street view map learning group (passive).

No significant differences were observed in terms of accuracy between the active learning group and the passive learning group [*t*_(__58__)_ = −1.914, *p* > 0.05, *d* = 0.49; *t*_(__58__)_ = −0.955, *p* > 0.05, *d* = 0.25; *t*_(__58__)_ = −0.848, *p* > 0.05, *d* = 0.22]. RT had a significant effect in the scene recognition task [*t*_(__58__)_ = 2.209, *p* < 0.05, *d* = 0.57], with the active learning group spending more time on the task, but differences in the other three tasks did not reach statistical significance [*t*_(__58__)_ = −1.643, *p* > 0.05, *d* = 0.42; *t*_(__58__)_ = 0.505, *p* > 0.05, *d* = 0.13; *t*_(__58__)_ = −0.028, *p* > 0.05, *d* = 0.01]. A significant effect was also observed in terms of accuracy, with the active learning group receiving lower scores [*t*(58) = −2.320, *p* < 0.05, *d* = 0.60] and no significant differences emerging in the remaining three tasks [*t*_(__58__)_ = −1.421, *p* > 0.05, *d* = 0.37; *t*_(__58__)_ = 0.348, *p* > 0.05, *d* = 0.09; *t*(58) = −0.866, *p* > 0.05, *d* = 0.22]. Regarding the absolute difference of distance task, no significant effect was observed [*t*_(__58__)_ = −0.451, *p* > 0.05, *d* = 0.12].

## Discussion

In summary, our results showed the observable impact of learning methods on spatial knowledge acquisition. The integrated analysis revealed that the 2D map-assisted field navigation learning is the relatively optimal learning method for spatial knowledge acquisition, followed by 2D map-assisted street view map passive learning. However, the effect of 2D map-assisted street view map active learning and field navigation learning is relatively inconspicuous.

Moreover, another issue is that the internal relationship among these four learning methods may not be simply parallel. For example, the accuracy and confidence ratings for the scene recognition task showed that the learning performance with street view map passive learning was close to that with 2D map-assisted field navigation learning, especially in landmark knowledge, and the two learning groups both performed better than the field navigation learning group. These results indicate that virtual learning is as suitable as physical learning is for acquiring analogous landmark knowledge. However, because two potential variables (i.e., virtual learning/physical learning, active learning/passive learning) exist between street view map passive learning and 2D map-assisted field navigation learning, concluding which factor is in effect is difficult. Therefore, on the basis of the results of pairwise comparisons, we specifically analyze the roles of different potential variables in acquiring spatial knowledge.

### Role of 2D Maps

Previous studies showed that maps play an important role in improving wayfinding performance. However, experiments of field navigation learning often examine whether individuals can find a route to their destination while failing to investigate the role of maps in the acquisition of spatial knowledge. Our findings imply that 2D map assistance in field navigation learning helps individuals acquire both configurational knowledge and landmark knowledge.

Rossano et al. (1999) separately analyzed the advantages of field navigation learning and map learning. They found that the field navigation learning group performed significantly better than the map learning group in route distance estimation, wayfinding, and other tasks, while the map learning group performed better than the field navigation learning group in line distance estimation and spatial orientation. In our study, results showed that the accuracy of the 2D map-assisted field navigation learning group was significantly higher than that of the field navigation learning group in scene recognition tasks, orientation judgment tasks, and configurational representation tasks. In terms of confidence ratings for each test, the effect was significant or marginally significant, with higher scores in the 2D map-assisted field navigation learning group. Similar results were found in their confidence ratings of correct answers, which means that the global map was able to effectively enhance participants’ self-confidence in spatial learning.

The results of this comparison are also consistent with the findings of existing studies (Thorndyke and Hayes-Roth, 1982; Richardson et al., 1999). In the field learning group, with a limited scope of observation, participants could develop memory from a local perspective only; the development of configurational knowledge requires mental integration of numerous continuous scenes from different perspectives, promoting high-level cognitive processing. Traditional 2D maps help learners understand the overall layout of an environment and gain spatial knowledge (e.g., spatial orientation and distance between landmarks). Arguably, a 2D map is an irreplaceable tool in terms of learning new environments.

The 2D map-assisted field navigation learning combines the advantages of field navigation learning and 2D map learning to compensate for their respective disadvantages. This feature enables individuals to develop not only configurational knowledge, but also knowledge of the representation of landmarks, routes, and other details. One’s environment can be a rich source of information, which means that the memory burden of learners can be heavy. A major challenge is that, through field navigation learning, learners can only code small pieces of spatial knowledge and cannot understand spatial layout as a whole; that is, configurational knowledge is poor. By comparison, 2D map learning lacks a specific sense of real environment, and representation of environmental details may be incomplete. Furthermore, materials, presentation ways, and other factors may influence the learning process with 2D maps.

While the two groups learned the environment within an identical time range (25 min), the 2D map-assisted field navigation learning group outperformed the field navigation learning group in terms of accuracy. Therefore, we conclude that 2D map-assisted field navigation learning is superior to field navigation learning in helping learners acquire spatial knowledge. In addition, our results prove the importance of maps’ role in the learning process, as they are particularly useful to learners for obtaining configurational knowledge and forming configurational representation more completely.

### Influence of Interaction Modes (Virtual Interaction or Physical Interaction)

Past studies surrounding these tasks focused on the difference in the performance of groups under different virtual environmental conditions and/or the comparison between transitioning from virtual environmental learning to field navigation learning and single field navigation learning. Clearly, a comparative study of the learning in VEs and physical environments is needed. One purpose of our experiment was to determine the difference between the two modes of interaction (virtual interaction or physical interaction) or learning and to verify whether virtual interaction can replace physical interaction. Our results confirm that 2D map-assisted field navigation learning showed an obvious advantage in accuracy and confidence ratings of all tasks.

The effect of virtual field navigation learning is largely determined by experimental equipment and the complexity of a new environment. Higher-immersion environments are conducive to learning, and lower-immersion environments limit learners’ acquisition of spatial knowledge. Czerwinski et al. (2002) found that when using a display device with a larger view range, female participants achieved similar navigation performance as male participants, thereby showing that the gender bias found in past reports disappeared. Boumenir et al. (2010) compared the learning effects of virtual navigation, physical navigation, and 2D maps and found that the physical navigation group could identify the same route multiple times, regardless of whether the route was simple or complex, while the virtual navigation group failed to reidentify complex routes. Therefore, because virtual display technology clearly presents limitations, differences between VE and physical environment learning lead to differences in spatial representation formed from either learning type. In addition, the effect of virtual field navigation learning is closely related to individual experiences (such as 3D game experiences), thereby affecting wayfinding and spatial representation processes.

Therefore, we argue that the main reasons for the virtual interaction group’s poorer performance are as follows:

(1) Our measurement methods differed greatly from those used in previous studies. Several cognitive factors were analyzed to identify the aspect that constituted the basis of spatial knowledge, and we argue that the validity of our evaluation is greater by comparison.
(2) The combination of 2D maps and the physical environment provided richer learning cues to learners. In the 2D map-assisted field navigation learning group, the participants could acquire survey knowledge through the map and through the scenes of different perspectives provided by the physical environment. More learning cues were available to the 2D map-assisted field navigation learning group; thus, the performance is correspondingly higher.
(3) The scale of the learning environment and the complexity of its information can impact learning. In large-scale and high-complexity environments, virtual street view learning cannot facilitate the acquisition of complete spatial knowledge, but small-scale and low-complexity environments are capable of doing so.

Virtual street view learning ultimately cannot replace field navigation learning. Even with the aid of 2D maps, virtual learning did not engender the same level of performance in tasks than field navigation learning did. Still, most researchers believe that VE is a good substitute for physical environments, and learning in VEs is as effective as in actual physical environments (Ruddle et al., 1997; Ohuchi et al., 2006; Lahav and Mioduser, 2008; Münzer and Zadeh, 2016). Virtual interaction (such as virtual tours) allows learners to navigate an unknown environment without having to go outdoors. Virtual street view map learning integrates the features of 2D map learning and field navigation learning. With the use of 3D technology, it retains a specific image of the physical environment while equipped with 2D maps. When learning the environment, users can adjust the scale of the 2D map or street view map at any time. This condition means that virtual interaction meets the needs of different learners. Virtual learning also excludes extraneous variables of field navigation learning, as the experimental environment can be artificially set up according to the purpose of study, thereby greatly reducing time, labor, and other costs (Snopková et al., 2019). Using a virtual model of the environment, Münzer and Zadeh (2016) efficiently investigated spatial knowledge acquisition in different training conditions. Moffat et al. (1998) used a 3D virtual environment to quickly explore the influence of sex differences on spatial ability. Overall, we can conclude that VEs give researchers new insight into what is happening during the process of wayfinding. Therefore, with respect to technology development, virtual interaction may gradually replace physical interaction. Future studies should address how we may narrow the immersion gap between virtual interaction and physical interaction and mitigate the disadvantages of virtual interaction.

Our results indicate that different modes of interaction greatly influenced the acquisition of spatial knowledge. For large-scale environments, field navigation learning is arguably more conducive to the acquisition of spatial knowledge. Meanwhile, the generalizability of our results is limited in the sense that this study was largely restricted to tasks and devices. With the popularization of VEs, virtual interaction will inevitably eventually replace physical interaction in the future. We argue that our focus should be on improving learning efficiency in VEs.

### Differences Between Active Learning and Passive Learning

The main difference between passive learning and active learning was that the former involved no interaction, whereas the latter was interactive. Although the results were not significant, passive learning still tends to be superior to active learning. The only exception in this part of the study occurred with the scene recognition and direction judgment tasks, in which passive learning did not yield better learning outcomes than active learning. Orientation judgment requires perspective transformation, while virtual learning lacks the mental and physical manipulation that field navigation learning allows (including motion control, proprioception, spatial updating, etc.). Without active interaction, the participants encountered difficulty engaging in mental manipulation, such as perspective transformation, during learning. This finding may explain why the passive learning group did not perform as well as the active learning group in these tasks. Overall, our results show no significant difference between the two learning methods in terms of influencing participants’ acquisition of spatial knowledge, and that, even in the scene recognition and configurational representation tasks, the passive learning group’s performance was superior to the active learning group’s performance.

These findings contradict Meade et al. (2019) assertion that, in VEs, active learning is more likely to result in higher accuracy of spatial memory than passive learning and that this effect is more significant in older adults. However, given the large gap between Meade et al. (2019) study and this experiment, new data may have affected our understanding of the learning environment and knowledge measurement paradigm. Previous research (Hazen Nancy, 1982; Peruch et al., 1995; Chrastil and Warren, 2013) contended that active learning groups outperform passive learning groups in wayfinding performance. One possible reason for this inconsistency is that these studies directly assessed the impact of learning modes on wayfinding ability rather than on spatial knowledge.

This comparison implies that interaction mode (passive learning or active learning) has little effect on the acquisition of spatial knowledge using street view maps. We argue that the main reasons for such results are as follows:

(1) The attention assignment in the two groups was different. The participants in the active learning group needed to assign part of their attention to the operation rather than the exploration and memorization process. This condition may have disrupted spatial learning performance. Comparatively, the passive learning group did not need to operate a map and simply watched the assigned video. This approach likely optimized the passive learning group’s input, reducing distractions and helping them to better develop spatial representation.
(2) Both active learning and passive learning were equipped with 2D digital maps, which may have caused an excessive contribution of spatial parallel processing in working memory. The contribution of mental manipulation in active learning is not very large, arguably leading to a disappearance of the SPT effect. Coluccia et al. (2007) showed that mental manipulation in working memory does not strongly contribute to active learning primarily because maps that provide parallel information about spatial layout play a fundamental role in learning.

Overall, the difference between passive learning and active learning is not considerably large, and some current research even argues that passive learning is better than active learning (Allison and Redhead, 2017). Our findings indicate that efficient passive learning using street view maps is possible and that passive learning is a viable replacement for active learning.

## Limitation and Future Work

In terms of the limitations of our study, the process of field navigation learning presented challenges in terms of different weather conditions, car traffic, and other uncontrollable factors that affected the participants’ performance. Future studies should consider how such extraneous factors can be reduced. In addition, the sense of dynamic change of the street view maps was not strong in virtual street view learning. Therefore, intelligent virtual reality systems that can accommodate this phenomenon should be considered in future research. Moreover, the 2D map used in the virtual street view learning is virtual, which may be different from the physical 2D map used in field navigation learning. Therefore, evaluating whether a 2D map is needed and whether a difference exists between having a virtual 2D map or a physical 2D map coupled with the virtual street view map in the future are meaningful.

Another potential issue may be the experimental design. We did not use a within-subjects design. If the within-subjects design was chosen, two coessential routes need to be selected to avoid the learning effect. Controlling the extraneous variables between two real routes is difficult. However, a within-subjects design may be more suitable to control the individual difference in spatial knowledge and observe more prominent differences among different learning methods. Although many studies (Meilinger et al., 2006; Parush et al., 2007) used between-subjects design while not measuring individual differences in spatial skills (our research did the same; an equal number of participants were assigned randomly to one of the conditions, which may decrease the variation among different groups), spatial ability measures of participants are needed to control the individual difference in spatial knowledge, thereby making the results more accurate and convincing. Therefore, a within-subjects design with counterbalanced learning method sequences is worth considering in the future.

Future studies should allow active learning groups to engage in route decision-making while controlling consistent visual input to provide more adequate active learning conditions (Guo et al., 2019). Virtual learning devices that provide physical information, such as omnidirectional treadmills, should also be considered for use in studies to verify the effect of physical information on spatial knowledge acquisition.

## Conclusion

In summary, this study compared the impact of virtual learning and field navigation learning on spatial knowledge acquisition, as well as the differences between active learning and passive learning by using virtual street maps. For the measurement of spatial knowledge, a set of dimensions were presented, which measured spatial knowledge such as scene recognition, orientation judgment, configurational representation, and distance representation. This study found that passive learning can replace active learning using street view maps, which has practical value for spatial learning and related research.

The following conclusions were drawn from this study:

(1) 2D map-assisted field navigation learning yields better outcomes than field navigation learning, which means that 2D maps play a significant role in facilitating individuals’ acquisition of configurational knowledge.
(2) 2D map-assisted field navigation learning yields better outcomes than active learning using street view maps. Physical interactive learning is also superior in terms of acquisition of landmark knowledge and configurational knowledge.
(3) No significant difference exists between passive learning and active learning using street view maps. When high-quality learning materials are provided, passive learning facilitates the acquisition of spatial knowledge more easily than active learning.

Field navigation learning is still the best way to acquire spatial knowledge. However, it entails large costs, and its complex physical environment causes logistical challenges. Virtual learning, such as street view map learning, alleviates those challenges. Furthermore, with high-quality VEs, individuals can form complete spatial knowledge that is close to the knowledge they would obtain via navigation learning. However, the cost of VE development is high and virtual active learning is limited by the form of learning, that is, a device can be used by only one learner in a certain period of time. Therefore, this learning method may not be suitable for a large number of learners to obtain spatial knowledge in a short period of time. Our findings show that providing learners with navigational videos of environments is the most economical and efficient method of spatial learning, especially when training a large number of learners.

## Data Availability Statement

The raw data supporting the conclusions of this article will be made available by the authors, without undue reservation, to any qualified researcher.

## Ethics Statement

The studies involving human participants were reviewed and approved by the Institutional Review Board (IRB) of the Zhejiang Sci-Tech University. The patients/participants provided their written informed consent to participate in this study.

## Author Contributions

LW and DW designed the study and analyzed the data. XQ, LW, CW, and ZY wrote the manuscript. LW participated in participants’ recruitment and data collection. XQ, LW, ZY, QW, HL, and DW participated in the data preprocessing and discussion. CW, ZY, and DW supervised the whole study.

## Conflict of Interest

The authors declare that the research was conducted in the absence of any commercial or financial relationships that could be construed as a potential conflict of interest.

## Footnotes

1 https://dwz.cn/4T7ExxYL
